# MRI T2 mapping and shear wave elastography for identifying main pain generator in delayed-onset muscle soreness: muscle or fascia?

**DOI:** 10.1186/s13244-024-01619-6

**Published:** 2024-02-29

**Authors:** Congcong Fu, Yu Xia, Bingshan Wang, Qiang Zeng, Shinong Pan

**Affiliations:** 1 Department of Magnetic Resonance Imaging, Zhongshan Hospital of Xiamen University, Xiamen, Fujian China; 2 Department of Medical Ultrasonic, Zhongshan Hospital of Xiamen University, Xiamen, Fujian China; 3 Department of Radiology, Shengjing Hospital of China Medical University, Shenyang, Liaoning, China

**Keywords:** Fascia, Muscle injury, Eccentric exercise, MRI, Ultrasound

## Abstract

**Introduction:**

The main generator of delayed onset muscle soreness (DOMS) is still unknown. This study aimed to clarify the main generator of DOMS.

**Methods:**

Twelve participants performed eccentric exercise (EE) on lower legs. MRI and ultrasound were used to assess changes of calf muscle and deep fascia before and after EE. These results were then compared to the muscle pain level.

**Results:**

Compared to baseline, muscle pain peaked at 24–48 h after EE (downstairs 22.25 ± 6.196, 57.917 ± 9.298, *F* = 291.168, *p* < 0.01; resting 5.833 ± 1.899, 5.083 ± 2.429, *F* = 51.678, *p* < 0.01). Shear wave speed (SWE) of the deep fascia and T2 values of the gastrocnemius muscle and deep fascia all increased and peaked at 48 h after EE (1.960 ± 0.130, *F* = 22.293; 50.237 ± 2.963, *F* = 73.172; 66.328 ± 2.968, *F* = 231.719, respectively, *p* < 0.01). These measurements were positively correlated with DOMS (downstairs: *r* = 0.46, 0.76, 0.87, respectively, *p* < 0.001; resting: *r* = 0.42, 0.70, 0.77, respectively, *p* < 0.001). There was a significant positive correlation between SWE and T2 values of deep fascia (*r* = 0.54, *p* < 0.01).

**Conclusion:**

DOMS is a common result of muscle and fascia injuries. Deep fascia edema and stiffness play a crucial role in DOMS, which can be effectively evaluated MR-T2 and SWE.

**Critical relevance statement:**

Delayed-onset muscle soreness is a common result of muscle and deep fascia injuries, in which the edema and stiffness of the deep fascia play a crucial role. Both MRI and shear wave elastography can be effectively used to evaluate soft tissue injuries.

**Key points:**

• The deep fascia is the major pain generator of delayed-onset muscle soreness.

• There is a significant correlation between fascia injury and delayed-onset muscle soreness.

• MRI and shear wave elastography are preferred methods for assessing fascia injuries.

**Graphical Abstract:**

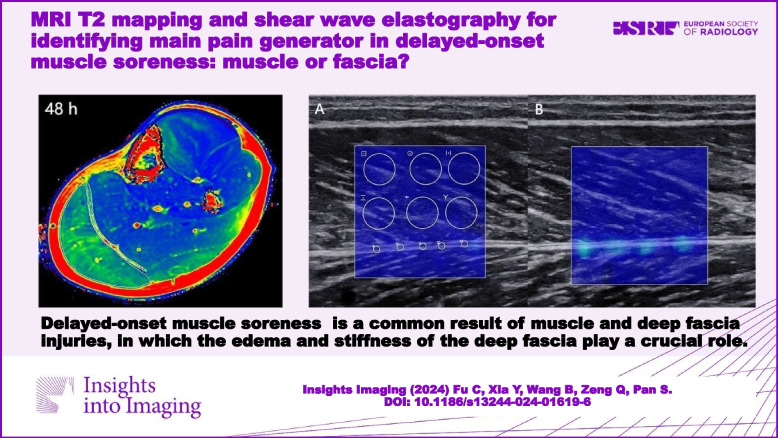

## Introduction

Eccentric exercises (EE) can result in delayed-onset muscle soreness (DOMS) [1–3]. In the “Munich consensus statement” [4], DOMS was classified as “muscle-related neuromuscular muscle disorder (type 1B)”. Many hypotheses to explain the mechanism of DOMS have been proposed and most studies have focused on the skeletal muscles [5–10]. However, there is increasing evidence to indicate that injuries to the fascial system could play a potential role in the development of DOMS. Thus, fascial tissues deserve more detailed attention [11–13].

The fascial system is a three-dimensional structure permeating the body that enables all body systems to operate in an integrated manner [13]. Pathological changes in the biomechanical properties of fascial tissues have been hypothesized to play a crucial role in musculoskeletal diseases [14]. The muscle fibers are closely fused with the endomysium in vivo, and the collagenous connective tissue is inextricably linked to the skeletal muscle [15]. Muscle fibers can transmit cross layer shear force through the fascia [16], and the fascia is very likely to be damaged during this transmission of force [17, 18].

The fascia comprises dense innervation, free nerve endings, and pain receptors, which make it more sensitive to pain [19–23]. By comparing the pain sensitivity of different structures to hypertonic saline, electrical stimulation, and local inflammation, some researchers were able to confirm that the fascia induced a stronger pain response than the muscle [24–26]. Thus, it can be speculated that the fascia, rather than the muscle, is the main generator of DOMS.

Imaging methods, such as high-field magnetic resonance imaging (MRI) and ultrasound shear-wave elastography (SWE), are promising tools for quantifying edema and hardness of the soft tissues under in vivo conditions [27, 28]. Especially, transverse relaxation time-weighted imaging (T2WI) is the most common approach [29]. As a parameter associated with osmotic changes, T2WI can provide information on the changes in water content of the muscle tissue, which can be quantified by T2-mapping technology. Due to the sensitivity of MR-T2 mapping to acute changes in the activity states of muscle, the method shows great potential for identifying muscle activation patterns in a variety of normal and pathological conditions [28, 30]. The results of previous studies have confirmed that T2 mapping can effectively and accurately evaluate the muscle damage caused by EE [28, 31, 32]. Second, the high-frequency ultrasonic probe produces an acoustic radiofrequency force impulse that generates transversely oriented shear waves that propagate throughout the surrounding tissues. The velocity of the propagating shear waves is measured on the qualitative color maps. Theoretically, the tissue elasticity can be calculated from the shear wave speed (SWS), providing important biomechanical information on tissue quality [33, 34]. However, due to its heterogeneity and anisotropic characteristics, SWE measurements in the musculoskeletal system should be presented in terms of the SWS in m/s [35, 36].

In the present study, MRI T2-mapping technology and SWE were applied to evaluate the edema and stiffness of the gastrocnemius muscle (GM) and deep fascia after EE, and to identify the main pain generator in DOMS.

## Methods

### Participants

Twelve healthy participants (6 men and 6 women; 18–30 years of age; body mass index = 19–24 kg/m^2^) were invited to participate in the prospective study. The inclusion criteria were as follows: no or little experience with weight-lifting exercises during the 90-day period before study participation, no basic diseases (cardiovascular, musculoskeletal, respiratory, or neurological diseases) that might affect the results. All participants were asked to avoid performing any sports activities for 1 week prior to the test and during the investigation period.

### Eccentric exercise

All participants performed a standardized EE of the calf muscles to induce DOMS. A specifically manufactured slant plate was set between the parallel bars, on which the participants performed EE after the warm-up. At the starting position, the participants raised their heels as high as possible, held this position for 1 s, and then lowered their heels slowly over 3 s until the sole touched the bottom of the slant plate. Participants pushed themselves up the parallel bars to move back to the starting position, allowing relief of the calf muscles. To increase the load, each participant wore a weighted backpack weighing approximately 20% of their body weight during exercise. All participants performed 4 sets of 12 repetitions, with a 30-s rest period between each set; however, the last three sets were performed until muscle fatigue occurred. Muscle fatigue was defined as obvious shaking of the leg and inability to control the speed of descent. In order to obtain accurate data, examinations were performed at approximately the same time of the day for each participant.

### Muscle soreness assessment

The level of muscle soreness was evaluated using a visual analog scale (VAS), with scores ranging from 0 (no pain) to 100 (worst pain). Muscle soreness was assessed before and at 0, 24, 48, 72, 120, and 168 h after exercise. The participants were asked to mark their muscle pain levels on the VAS scale at rest and while walking downstairs.

### Serum creatine kinase (CK) level

Blood (5 mL) was collected from the antecubital vein of each participant and centrifuged at 1000 g for 10 min. The serum CK levels were measured before and 24 and 48 h after the EE using the BECKMAN Au5800 automatic biochemical analyzer (Beckman Coulter, Inc, Bria, CA, USA).

### Magnetic resonance imaging

**Fig. 1:**
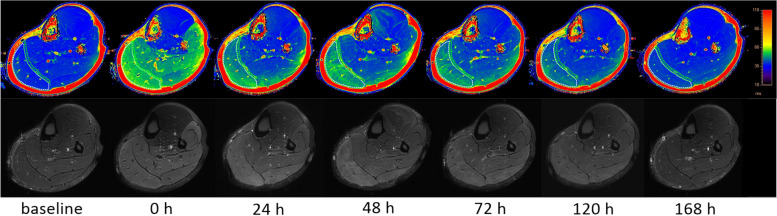
**a** T2 maps. **b** Fat-suppressed T2W images. **a** and **b** show the calf muscle before and after eccentric exercise; the data are measured on T2 maps. The color bar of the T2 maps ranges from 10 to 110

MRI scans were performed on a 3.0 T whole-body MR scanner (Ingenia CX, Philips Healthcare, Best, Netherlands) using a dedicated sensitivity encoding knee coil. Participants were positioned in the supine position. Both ends of the lower limb were raised to avoid compressing the calf muscles. Fat suppressed T2WI and T2 mapping were performed for the entire GM belly. T2-mapping scans were acquired as multi-shot SE sequences, echoes = 6; TE = 7.9, 15.8, 23.7, 31.6, 39.5, 47.4 ms; TR = 1006 ms; NAX = 1; flip angle 90°; FOV 160 × 160 × 75 mm; voxel 0.9 × 1.12; slice thickness 5 mm; interslice gap 0 mm. The original images were entered into the IntelliSpace Workstation (Philips), and the T2 values were automatically computed from T2 maps. The regions of interest (ROI) were drawn manually, and the borders of the ROI were traced to exclude the bone and large blood vessels. The ROIs were reconfirmed on the corresponding fat suppressed T2WI images. Three continual typical images of the GM belly were selected from the transaxial MR images. The final result was determined as the mean of the measured values in the above three images (Fig. 1). T2 values were assessed before and at 0, 24, 48, 72, 120, and 168 h after exercise.

### Ultrasound

**Fig. 2:**
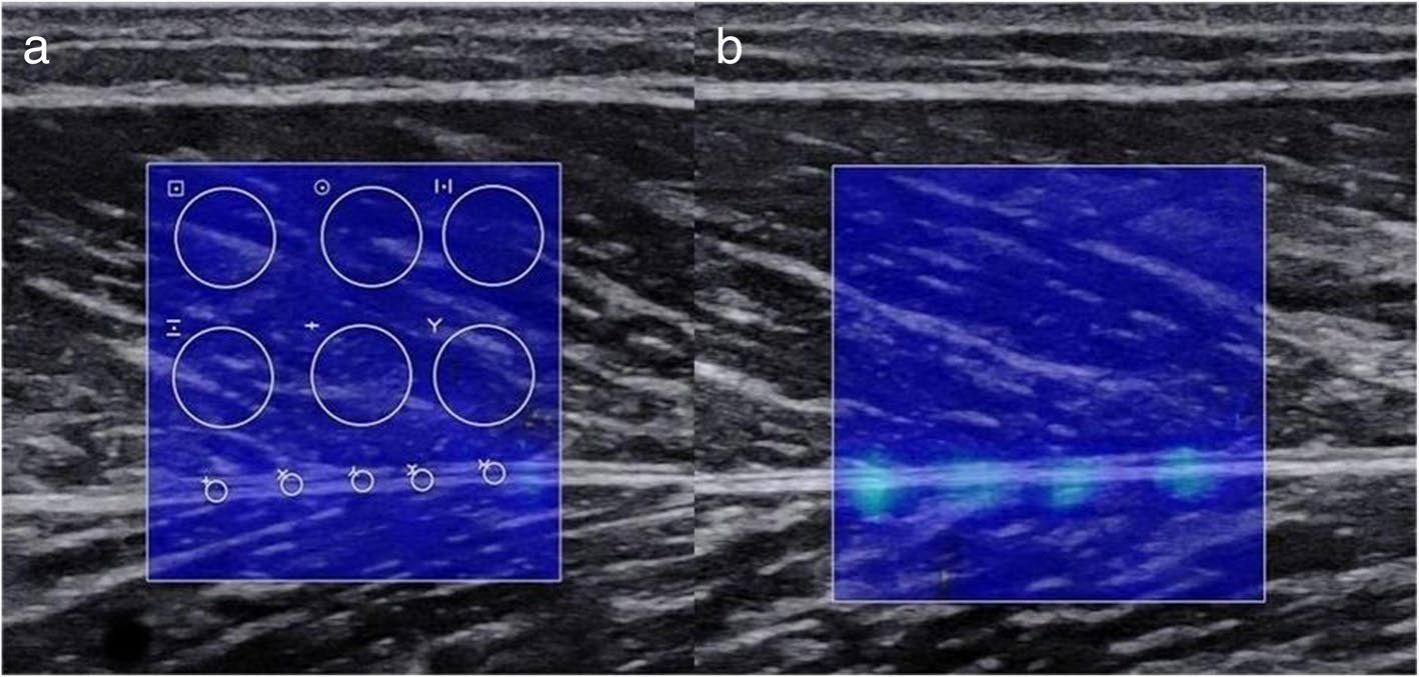
**a** and **b** separately show the shear wave elastography qualitative color maps of the lower leg soft tissue before and 48 h after exercise. Six regions of interest (diameter = 5 mm) were manually located at the medial head of the gastrocnemius muscle, while five regions of interest (diameter = 1 mm) were manually located equidistantly along the deep fascia

SWE was performed by one examiner educated in musculoskeletal ultrasound imaging with 10 years of work experience. The SWS (m/s) values of the GM and deep fascia were measured using a high-resolution ultrasound device with an 8.0 × 1.5 cm linear array transducer (frequency range of 10–2 MHz, display depth of 3.0 cm, dynamic range of 60 dB, and image gain of 50). To avoid false measurements caused by muscle tension, all participants were positioned in the prone position. A cushion was placed under the foot to keep the calf muscles relaxed, as muscle tension can significantly change SWS. To avoid compressing the tissue, the doctor stabilized the probe on the skin with minimal pressure. A sufficient amount of ultrasound gel was applied between the skin and the probe. To ensure identical positioning during the entire study, the probe location was marked on the calf skin with a semi-permanent marker. The transducer was positioned at the belly of the medial head of GM, perpendicular to the skin, avoiding interfering signals caused by the large vessels. A sagittal examination plane was chosen parallel to the muscle fibers to make the shear waves propagate better [37, 38]. To measure the SWS of the GM and deep fascia, six ROIs (diameter = 5 mm) were manually located at the middle of GM, while five ROIs (diameter = 1 mm) were manually located equidistantly along the deep fascia. The final results were determined as the mean of the measurements (Fig. 2). SWS values were assessed before and at 0, 24, 48, 72, 120, and 168 h after exercise.

### Statistical analysis

All data were tested for normal distribution, and inference statistics were applied as appropriate. For continuous variables that conform to a normal distribution, the mean ± standard was used for statistical description, while for those that do not conform to a normal distribution, the median (quartile) was used. Repeated measurement analysis of variance was used to compare CK levels at baseline and 24 h and 48 h. Before applying analysis of variance (ANOVA) of single factor repeated measurement data to check the trends in the changes of the degree of muscle soreness, T2 values, and deep fascia thickness, the Mauchly test was applied to verify sphericity. If *p* values > 0.05, it was considered that the data satisfies the sphericity assumption; one-way ANOVA was performed directly. Otherwise, the Greenhouse–Geisser correction was applied. Bonferroni’s multiple comparison was used for post hoc comparison.

Linear correlation analysis was performed to analyze the relationship between muscle injury, fascia injury, and DOMS, with T2 and SWS values as the independent variables and muscle pain level as the dependent variable. Linear correlation analysis was performed to analyze the relationship between edema and the hardness of soft tissue, with T2 values as the independent variables and SWS values as the dependent variable. Data were considered significant for *p* values < 0.05.

## Results

### Delayed-onset muscle soreness

**Fig. 3:**
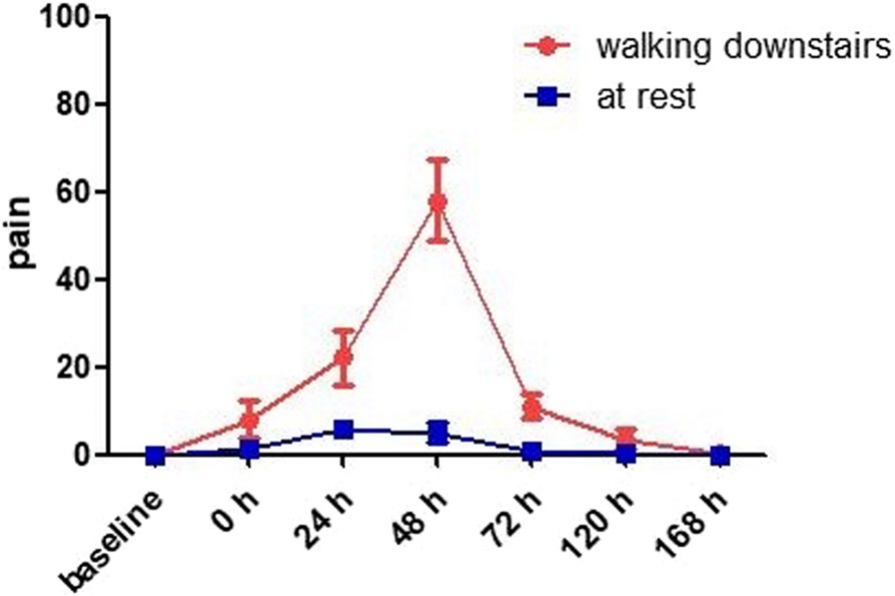
Pain measured on the visual analog scale (VAS 0–100): temporal changes in mean pain level before and after eccentric exercise (walking downstairs and at rest)

DOMS was successfully induced in each participant, and the changes in pain levels of the calf muscles are shown in Fig. 3. The results of one-way repeated measures ANOVA showed that, compared with the baseline value (0 ± 0), there was a significant difference in muscle soreness indicators of participants both when walking downstairs (0–168 h, 7.917 ± 4.316, 22.250 ± 6.196, 57.917 ± 9.298, 11.000 ± 2.828, 3.583 ± 2.021, 0.250 ± 0.452, *F* = 291.168, *p* < 0.001) and at rest (0–168 h, 1.25 ± 0.866, 5.833 ± 1.899, 5.083 ± 2.429, 0.667 ± 0.651, 0.333 ± 0.492, 0 ± 0, *F* = 51.678, *p* < 0.001). The results of Bonferroni’s multiple comparison showed that when walking downstairs, the muscle pain level at 0, 24, 48, 72, and 120 h after EE was significantly higher than the baseline level (separately, *p* < 0.01), reaching a peak at 48 h. The muscle pain had disappeared in all participants by 168 h after EE, there was no significant difference compared to baseline (*p* > 0.05). Conversely, in the resting state, all participants could feel a slight muscle soreness only at 0, 24, and 48 h after EE (separately, *p* < 0.01).

### Serum CK level

The results of one-way repeated measures ANOVA showed that, compared with the baseline value, there were statistical differences in serum CK level after EE (*F* = 10.88, *p* = 0.007). The results of Bonferroni’s multiple comparison showed that the serum CK level at 24 and 48 h after EE were significantly higher than the baseline level before exercise (separately, *p* = 0.024 and *p* = 0.018) (Table 1).

**Table 1:** Serum CK level of participants before and at 24 and 48 h after eccentric exercise

Group	Serum CK (*x̄* ± SD)	*F*-test	
		*F*	*p*
Baseline	95.53 ± 34.73	10.88	0.007
24 h	1087.96 ± 1080.88		
48 h	1053.85 ± 995.51		

*CK* creatinine kinase, *F-test* Fisher test

### Magnetic resonance imaging

**Fig. 4:**
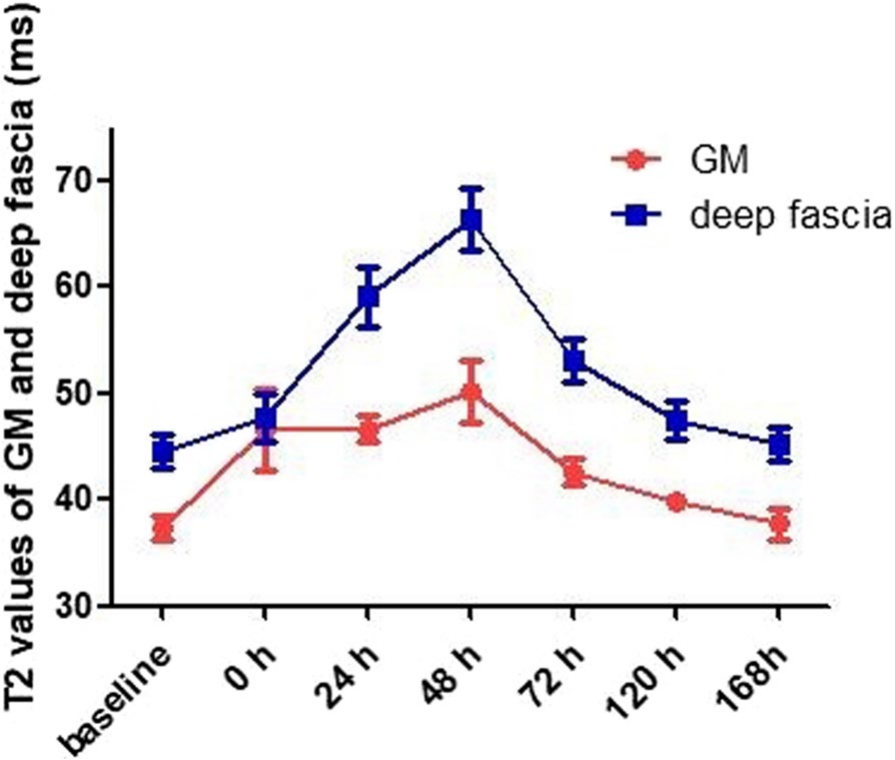
Temporal changes in the mean T2 values of the gastrocnemius muscle and deep fascia before and after eccentric exercise

The temporal changes in GM and deep fascia edema in all participants are shown in Figs. 1 and 4. Under physiological conditions, no signs of edema were observed in any part of the calf muscles or deep fascia. The T2 values of the GM increased immediately following EE, peaking at 48 h. Subsequently, the signal intensity and range decreased over time. During this period, the results demonstrated muscle edema only after EE, with no architectural disorganization. The results of one-way repeated measures ANOVA showed that there were statistical differences in T2 values of GM after EE (0–168 h, 46.538 ± 3.832, 46.666 ± 1.153, 50.237 ± 2.963, 42.586 ± 1.283, 39.811 ± 0.757, 37.694 ± 1.436, *F* = 73.172, *p* < 0.001) compared with the baseline value (37.441 ± 1.138). The results of Bonferroni’s multiple comparison showed that the T2 values of GM at 0, 24, 48, 72, and 120 h after EE were significantly higher than the baseline level before exercise (separately, *p* < 0.01).

The results show that the increase in the T2 value of the deep fascia is obviously lower than that of the GM during the first 24 h. Representative fascial edema peaked 24 h after EE. The results of one-way repeated measures ANOVA showed significant increases in the T2 values of the deep fascia after EE (0–168 h, 47.679 ± 2.216, 59.114 ± 2.790, 66.328 ± 2.968, 53.058 ± 2.057, 47.519 ± 1.831, 45.219 ± 1.528, *F* = 231.719, *p* < 0.001) compared with the baseline value (44.573 ± 1.636). The results of Bonferroni’s multiple comparison showed that the T2 values of the deep fascia at 0, 24, 48, 72, and 120 h after EE were significantly higher than the baseline level (separately, *p* < 0.01).

At 168 h after EE, there was no significant difference in T2 values of GM and deep fascia compared to baseline (*p* > 0.05).

### Ultrasound

**Fig. 5:**
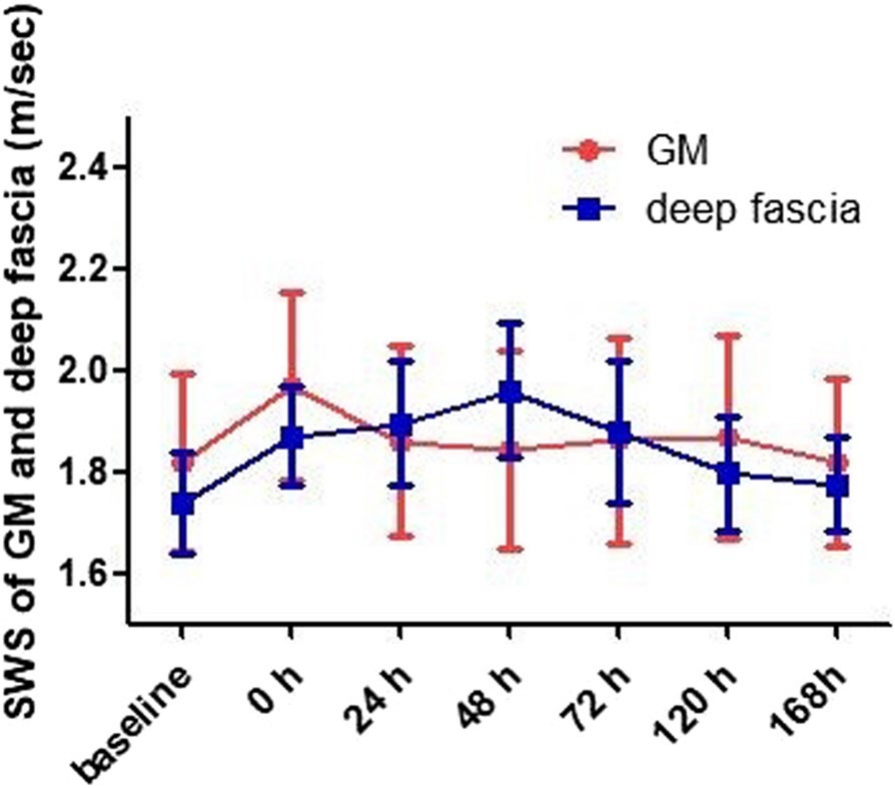
Temporal changes in the mean shear wave speed of the gastrocnemius muscle and deep fascia before and after eccentric exercise

The temporal changes of the SWS of the GM and deep fascia in all participants are shown in Figs. 2 and 5, with no architectural disorganization. SWS detected by ultrasound showed that the hardness of the GM rapidly increased after EE. The results of one-way repeated measures ANOVA showed that, compared with the baseline value (1.818 ± 0.177), there were significant increases in the SWS of the GM after EE (0–168 h, 1.967 ± 0.185, 1.860 ± 0.187, 1.842 ± 0.195, 1.862 ± 0.204, 1.868 ± 0.199, 1.818 ± 0.166, *F* = 9.947, *p* < 0.001). The results of Bonferroni’s multiple comparison showed that the SWS of the GM was higher than the baseline level at 0 h after EE (*p* = 0.024).

The SWS of the deep fascia increased after EE, peaking at 48 h. The results of one-way repeated measures ANOVA showed that, compared with the baseline value (1.740 ± 0.100), there were statistical differences in the SWS of the deep fascia after EE (0–168 h, 1.870 ± 0.098, 1.896 ± 0.120, 1.961 ± 0.130, 1.878 ± 0.138, 1.798 ± 0.113, 1.775 ± 0.092, *F* = 22.293, *p* < 0.001). The results of Bonferroni’s multiple comparison showed that the SWS of the deep fascia at 0, 24, 48, and 72 h after EE were significantly higher than the baseline level before EE (separately, *p* < 0.05). At 120, 168 h after EE, there was no significant difference in SWS of deep fascia compared to baseline (*p* > 0.05).

### Correlation

**Fig. 6:**
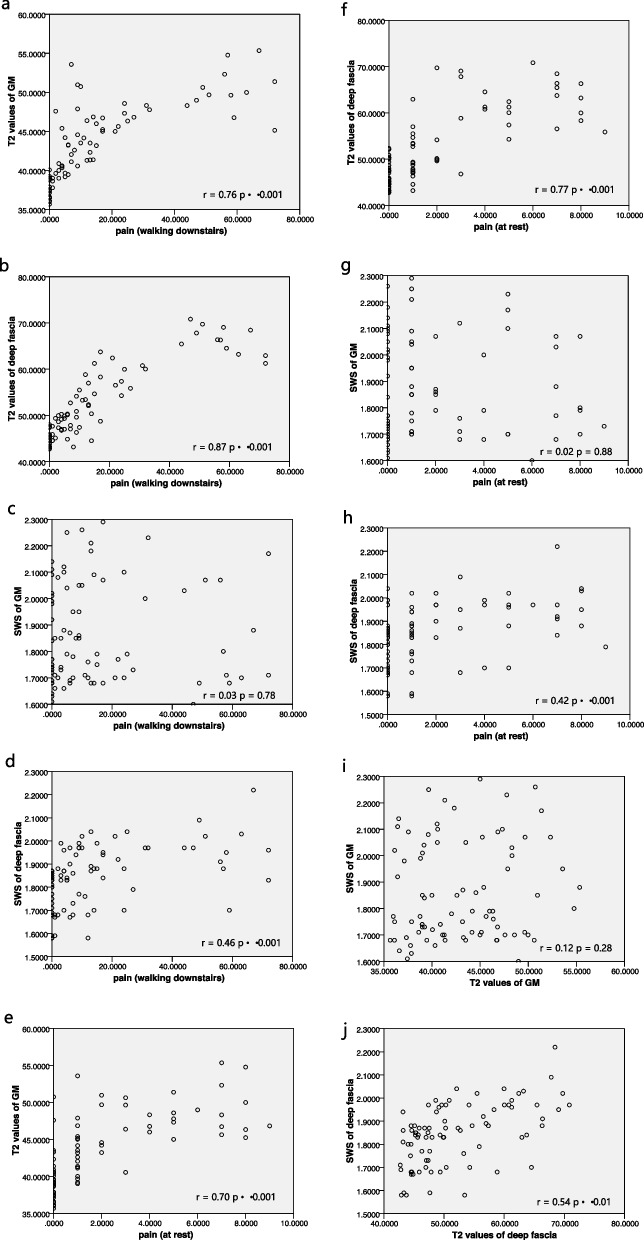
**a**, **b**, **e**, and **f** show the correlation between the pain level (when walking downstairs and at rest) and the T2 values of the gastrocnemius muscle and deep fascia; **c**, **d**, **g**, and **h** show the correlation between the pain level (when walking downstairs and at rest) and the shear wave speed of the gastrocnemius muscle and deep fascia; **i** and **j** show the correlation between the T2 values and SWS

The linear correlation analyses results are shown in Fig. 6. Linear correlation analysis showed that muscle pain level (downstairs and at rest separately) correlated strongly with T2 values of GM (*r* = 0.76, *p* < 0.001 and *r* = 0.70, *p* < 0.001) and T2 values of deep fascia (*r* = 0.87, *p* < 0.001 and *r* = 0.77, *p* < 0.001) and correlated with SWS of deep fascia (*r* = 0.46, *p* < 0.001 and *r* = 0.42, *p* < 0.001) following EE. There was no significant correlation between muscle pain level (downstairs and at rest separately) with SWS of GM (*r* = 0.03, *p* = 0.78 and *r* = 0.02, *p* = 0.88). In addition, T2 values of deep fascia was positively correlated with SWS of deep fascia (*r* = 0.54, *p* < 0.01). There was no significant correlation between T2 value and SWS of GM (*r* = 0.12, *p* = 0.28).

## Discussion

In this study, all participants successfully induced DOMS, with pain peaking at 48 h after EE, both when walking downstairs and resting. The muscle injuries of all participants were confirmed as type 1B [4]. The SWS of the deep fascia and the T2 values of both the GM and deep fascia showed positive associations with DOMS. Meanwhile, there was a significant correlation between T2 values and the SWS of the deep fascia. These results suggested that edema of both the muscle and deep fascia may be involved in the development of DOMS. Due to the structural characteristics of the fascia and its high sensitivity to injury and inflammatory stimulation [13, 19–21], we speculated that the deep fascia would play a crucial role in DOMS. In this study, we produced strong evidence to support this hypothesis, but there are still several details that need to be discussed.

### Before EE

According to previous studies, damage to the GM after EE is more obvious than in other calf muscles [38, 39]. In addition, due to limitations in the SWE detector, it is difficult to detect deep muscles and accurately measure changes in their hardness [40, 41]. Therefore, GM and its adjacent fascia were selected as observation objects. The serum CK level was used as an indicator of muscle injury in this study. Many factors, including training mode, age, and gender may significantly influence CK levels [42, 43]. Furthermore, serum CK activity after exercise is poorly related to muscle soreness level, strength, and range of motion [44]. Therefore, CK appears to be of more use as a qualitative marker, rather than a quantitative indicator, of muscle damage [42].

### Immediately post-EE

The results showed that the T2 values of the posterior and lateral groups of calf muscles increased significantly. The T2 signal is determined through the metabolism of water molecules in the muscle, while an increased relaxation time of skeletal muscles reflects tissue activity-related changes [28]. T2 values from T2 maps can be used as a quantitative index of changes in tissue composition, water metabolism, and other biochemical changes, implying a significant increase in calf muscle perfusion following EE [45], but without obvious architectural disorganization. In contrast, the increase in the T2 values of the deep fascia is obviously lower than that of the GM, indicating that no apparent edema or inflammatory exudation occurred in the deep fascia at this stage. In addition, representative fascial edema peaked 24 h after EE. These results are similar to previous conclusion [1, 3, 6, 46] and may provide a reasonable explanation as to why DOMS typically presents 24–72 h after EE. In the present study, many of the participants felt only discomfort, rather than pain immediately following EE. This may be due to the fact that regular and acute exercise can increase water content and muscle volume [47], leading to an increase in fascia tension [6, 10, 45, 46, 48]. Therefore, we speculate that this is the main reason for discomfort at this stage.

The relative increase in deep fascia hardness at 0 h after EE was observed from the SWE results, confirming the above hypothesis [34, 36]. However, this hypothesis still requires further verification. In the future, more tests should be conducted 0–24 h after EE to study what happens to the muscles and fascia on the first day.

### Degeneration processes

The CK results at 24 and 48 h after EE showed that all of the participants successfully induced delayed injury to GM.

The MRI results demonstrated that obvious injury and inflammatory exudation occurred in GM based on uneven signals [31]. With reference to MRI, bound water shows a shorter relaxation time than free water [49], and free water in the intermuscular space is rare. Therefore, normal muscle tissues exhibit an even shorter T2 signal intensity [50]. According to the previous study [31], the distributions and states of intracellular and extracellular water were altered by the inflammatory reactions following EE. After EE, the muscle fibers were arranged irregularly, and the extracellular spaces were enlarged, with inflammatory cells gradually infiltrating and gathering. The content of bound water in the intercellular space increased. The permeability of skeletal muscle cells increased, and large molecules in cells entered the extracellular fluid, resulting in a decrease in the bound water on the cellular surface. Meanwhile, free water from the interstitial space entered muscle cells, leading to cellular swelling. These changes prolonged the relaxation time and affected the uniformity of the image.

The SWS and T2 values of the deep fascia increased significantly from 24 to 48 h after EE. Recent results showed that both the thickness and hardness of deep fascia increases after EE [18, 51]. These findings indicate that fascial micro-injury and edema may be potential causes. Earlier studies have shown that edema is associated with increased stiffness [6, 52, 53]. Our findings confirmed a moderate positive correlation between fascial edema and stiffness, providing some support for their hypothesis.

In clinical practice, muscle injury rarely occurs alone, and injury to the collagen connective tissue is very common in exercise-induced muscle strain [17, 54]. Muscle fibers are tightly fused with the endomysium in the body, and collagen connective tissue is inseparable from skeletal muscle [15, 55]. They can transmit force between both ends of the muscle through the endomysium [16]. EE generates high shear force, which may damage the fascia and cause edema due to the mechanical interaction between adjacent muscle fibers [17, 18]. This overstretch could cause micro damage in the fascia, which may contribute to the development of fascia edema post-exercise. Our results show that, compared with the muscle, deep fascia injury has a higher correlation with DOMS. According to the high sensitivity of the deep fascia to edema and inflammation [19–26], we believed that both muscle and fascial edema contribute to DOMS; however, the deep fascia seems to be the main generator of DOMS at this stage.

In our study, SWE was used to evaluate the hardness of soft tissue. Compared to the baseline values, the hardness of the GM is significantly higher than that immediately after EE. There was no significant correlation between the hardness of GM and DOMS. These results largely, but not completely, agree with those of previous studies [56, 57]. We speculated that one reason to explain our finding may be that different muscles have different responses to EE, and the influence of muscle tissue viscoelasticity, heterogeneity, and anisotropy cannot be ignored [35, 36]. Furthermore, muscle quality and structure may further affect the recovery rate after resistance training [58, 59]. In addition, the daily use rate of the leg muscles is significantly higher than that of the arms, which may promote quicker recovery [60]. To sum up, we confirmed that a single bout of EE does not affect muscle stiffness constantly, and there is no significant correlation between muscle stiffness and DOMS. We therefore believe that muscle stiffness cannot be used to explain DOMS yet.

### Regeneration processes

The magnitude of DOMS decreased gradually after peaking at 48 h post-EE, and the pain had disappeared in all participants by 168 h after EE. The MRI results showed that the edema of the GM and deep fascia declined gradually and recovered to baseline at 168 h after EE. With the disappearance of edema, the hardness of the deep fascia gradually recovered, returning to the baseline level at 120 h.

### Limitations

Firstly, the sample size in this study was small. This study was only prospective; in future research, we will consider expanding the sample size and diversity of research objects. Secondly, due to the small sample size of this study, it is not possible to establish cut-off values to differentiate normal muscle and fascia from DOMS. In addition, multiple examinations within 0–24 h should be added, which will help to understand the details of the muscle and fascia evolution in the early stage of injury. Furthermore, more muscles will be included in future research.

## Conclusion

In conclusion, we confirmed that the deep fascia, rather than the muscle, is the predominant pain generator of the complicated process of DOMS. The changes in MRI T2 values and ultrasound SWS values can accurately display the edema and hardness of the deep fascia. Due to non-invasive and quantitative characteristics, T2 mapping and SWE could become preferred methods for assessing soft tissue injuries in DOMS.

## Data Availability

The datasets used and/or analyzed during the current study are available from the corresponding author on reasonable request.

## Abbreviations

DOMS: Delayed-onset muscle soreness
EE: Eccentric exercise
MRI: Magnetic resonance imaging
SWE: Shear-wave elastography
SWS: Shear wave speed
